# The Thermal Properties of a Prototype Insulation with a Gyroid Structure—Optimization of the Structure of a Cellular Composite Made Using SLS Printing Technology

**DOI:** 10.3390/ma15041352

**Published:** 2022-02-12

**Authors:** Beata Anwajler

**Affiliations:** Faculty of Mechanical and Power Engineering, Wroclaw University of Science and Technology, Wybrzeze Wyspianskiego 27, 50-370 Wroclaw, Poland; beata.anwajler@pwr.edu.pl

**Keywords:** 3D printed, thermal insulation, closure, structure, TPMS

## Abstract

This paper focuses on the search for novel insulating structures, and the generation of them by means of a state-of-the-art manufacturing method—3D printing. Bionic structures, which are successfully used in many branches of technology, were chosen as the source of inspiration for the research. The paper presents a design of spatial structures with a gyroid infill (e.g., TPMS), the shape of which reflects the bionic structure of the inside of a bone. For SLS printed single- and multi-layered structures, the design value of the thermal conductivity coefficient was determined through measurements and calculations. A statistical analysis was carried out to determine the effect of the direction of heat flow, as well as the internal structure and layering of the prototype materials, on the values of the thermal conductivity coefficient and the thermal resistance coefficient. On the basis of the multicriteria analysis, the composite’s optimal composition according to the adopted optimization criteria was determined. The lowest possible thermal conductivity of the insulation was equal to 0.033 W/(m·K). The highest possible thermal resistance was equal to 0.606 m^2^·K/W. Thermal insulation made of the prototype insulating partitions with a gyroidal structure is characterized by good insulating parameters.

## 1. Introduction

As the demand for electricity from the National Power Grid (NPG) increases, new ways of reducing this demand are being sought. The peak demand for electricity from the NPG in Poland coincides with the occurrence of low outdoor temperatures. In new build projects, a great emphasis is put on limiting heat losses (in the heating season) and heat gains (in the non-heating season), whereby energy expenditures on cooling and heating can be minimized. Typical measures adopted for this purpose include the use of insulation with both a low thermal conductivity coefficient and a special window frame design [1,2]. The thermal properties of windows are critical for the high thermal performance of buildings. Windows are generally responsible for about 30–50% of the heat transmission losses of a building, even though the share of the building’s shell area is considerably smaller. In the case of old builds, it is not possible to freely use measures that limit heat loss. The thermal upgrading of such buildings mainly involves the insulating of roofs and walls with a material that exhibits high heat conduction resistance. This is carried out in places where such insulation was not formally present, or where the previous material was characterized by unsatisfactory parameters. The need for effective thermal insulation is not solely the domain of the construction business, with an example of this being cold chambers, which are understood as both household fridge freezers and industrial cold stores. The desire to improve the energy performance of equipment and buildings, which can bring substantial environmental and economic benefits, as well as the performed analysis of the thermal insulation solutions that are currently available on the market, encouraged the search for novel insulating materials and the improvement of existing ones [1].

Bionics, as a science that analyses how living organism function, and the implementation of derived solutions in technology, has recently seen a rapid development. Bionic structures can ensure favorable operating parameters for equipment, such as high mechanical durability at a low specific weight, and it is therefore not surprising that design engineers readily use these kinds of solutions. 3D printing opens up new possibilities for rapid prototyping. Thanks to the multitude of 3D printing solutions, which make it possible to print almost any shape—through, e.g., melted material deposition, resin curing, or laser powder sintering—engineers are able design any model, as well as print and test specimens. A structure can be so complex that it is possible to be made in no other way than by 3D printing. Man-made insulations existing to date are characterized by a less complex structure than naturally occurring composites [3,4,5]. Bionics focuses on, i.a., the search for lightweight and durable materials. As an example, it is worth mentioning here the structure of a bamboo stalk. Bamboo is a plant that can reach a height of 20 m while still being able to keep its vertical position. It is interesting to note that bionic structures characterized by high strength usually have a cellular or fibrous structure. Another example of a structure characterized by both high strength and porosity is the structure of the inside of a bone. As the skeleton’s members are the load-bearing parts of living organisms, they must be able to transfer heavy loads while being lightweight, and it can therefore it be concluded that bones have high flexural and compressive strength [5,6,7].

**Figure 1:**
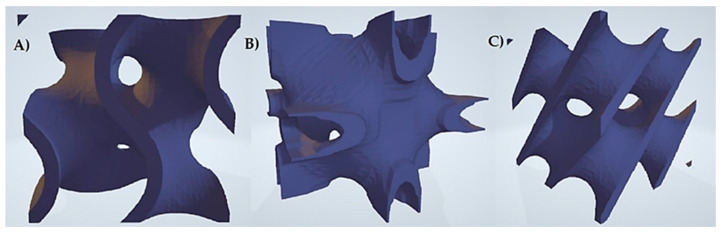
Elementary cells with TPMS architecture: (**a**) Gyroid, (**b**) Neovilius, (**c**) Schwarz-D.

This paper focuses on the state-of-the-art techniques referred to as 3D printing. In today’s world, designing a honeycomb shaped structure does not seem to be a big engineering challenge. However, not all bionic structures have such a simple pattern. There are shapes that are not regular and which reach a high degree of surface development. An attempt to model such structures with the use of basic geometric figures, the so-called geometric primitives, can turn out to be unfeasible. An interesting discovery is the generation of spatial structures called minimal surfaces, which are surfaces that reach the smallest possible area for the set boundary conditions [3,4,5]. A subgroup of minimal surfaces are triply periodic minimal surfaces (TPMS). These are characterized by shape repeatability in all three directions along the periodicity direction. TPMS structures are usually described with trigonometric functions, and therefore it is easy to deduce that the period of repeatability of such a structure depends on the kind of function. Due to the above property, a single block of a TPMS structure can be repeatedly duplicated while maintaining the structure’s continuity at the junctions between walls. Figure 1 shows a few popular periodic minimal surfaces [5,8].

The first TPMS structures were discovered towards the end of the 19th century. However, minimal surfaces had been in the range of interest of mathematicians since at least the previous century. Interestingly, analogies to these convoluted structures have begun to be found in nature. One of the more conspicuous similarities is the conformity between the inside structure of a bone and the surface of a gyroid. The two shapes are characterized by an open cellular nature and a visual similarity with regards to their surface pattern. When using triply periodic minimal surfaces, it is possible to build lightweight scaffolds and heat exchangers with a complex geometry, and also to create energy absorbing materials. An MIT (Massachusetts Institute of Technology) team designed one of the most durable lightweight materials known to man, and then simulated three-dimensional TPMS structures made of this material. The gyroid was found to have unusual mechanical properties. The results of tension and compression tests showed that the structure could be 10 times stronger than steel, but at the same time it was considerably lighter [9]. In [10], the authors showed that TPMS structures have excellent mechanical properties due to their isotropic smooth surface transitions and connectivity with open cells. Heat-resistant load-bearing elements are common in airplanes and should be characterized with lightness and high mechanical properties, which would in turn allow designers to achieve weight savings when designing new aircraft. In study [11,12], an elementary Schoen gyroid cell was used to design cellular structures. The TPMS structures exhibited optimal thermal and electrical conductivity [13]. Recent tests [14,15,16,17] have shown that the co-continuity of TPMS structures ensures stress transmission and plastic strain division mechanisms, in turn enabling continuous composites to withstand and take on greater strains before undergoing more serious damage. The test results also showed the superiority of structures based on TPMS over traditional conventional high mass constructions. Designing cellular structures with a triple period minimum surface area is a novel approach to lightweight and multifunctional structural applications [10]. Additive manufacturing, due to its inherent properties—i.e., freedom of manufacturing and layer after layer construction—has huge potential in the area of building such TPMS structures [12]. 3D printing technology is still relatively young and has many limitations, but the expectations and hopes for the future of 3D-printed buildings and building elements are high. Research concerning the versatile applications of 3D printers and the development of new filamentous materials that could provide various properties ensuring transparency, thermal insulation, or strength are being conducted [17,18,19]. Layered composites, considered as special materials, are widely used in the aviation, marine, automotive, and construction industries, and thanks to 3D printing technology they acquire a new meaning. Composites with the so-called sandwich structure [18], which have a low-density core and rigid layers that are arranged on the outside of the composites, are very often used. The core can be made in the form of closed, or open foam or periodic, structures [18]. Layered materials can be divided into four types: cellular foam, honeycomb, corrugated cardboard, and balsa [18].

Light sandwich panels are now widely used due to their high flexural stiffness to weight ratio, excellent thermal insulation, and high energy absorption capacity [18]. In literature [20,21,22,23], the authors use additive manufacturing to create layered structures with architectural cores. They showed, among other things, that the properties of cellular materials are not only determined by solid components, but also by the spatial configuration of voids and solids, i.e., cellular architecture. The change of the cellular architecture gives unlimited possibilities of obtaining the desired properties of materials [24].

3D printing is a promising new technology for erecting buildings. New prototype structures are currently being created all over the world using this method. 3D printing is considered to be a technology that can be used to print structures in an auto-mated manner on the Moon or Mars. The execution of building structures is associated with considerable expense and a long duration of performed work. Attempts to find a way to reduce the impact of these two factors include interest in 3D printing by entrepreneurs from the construction industry [17].

At the industrial level, technology has evolved in different directions—there are advanced materials, better quality, larger workspaces, and new additive technologies, including 3D concrete printing technologies. When talking about the 3DCP industry, everyone thinks about large constructions and buildings; however, it should be re-membered that 3D printing with concrete also incudes, and maybe even primarily, the erection of buildings and building objects. While the 3D printing of houses is still a distant subject, the printing of prefabricated elements (manholes, chambers, supports, columns), utility architecture (benches, tables, fountains), or decorations (monuments, facade decors, stucco) is very achievable even now. In this form, 3DCP technology is already starting to establish itself on the market [25].

It is assumed that the use of 3D printing in the construction industry may shorten construction time in the future. This would be due to the fabrication of elements on the construction site, which would reduce the intensity of transport to the construction site. An additional interesting aspect when designing would be the possibility of creating external facades with unusual shapes, which are still sought and desired in cellular architecture [17,25].

## 2. Materials and Methods

The range of possible applications of 3D printed insulating structures is wide. Such constructions can be filled with air or other gases—e.g., argon—and there can be a vacuum inside them. These materials can also be used in the electronic industry for the hermetic enclosures of devices, in the aviation industry as insulation in flat plate solar collectors, and in the food industry as bulk frozen food containers.

For the last several years, the author has been conducting research aimed at ob-taining an effective insulating material structure [1,26,27,28,29]. This paper is the continuation of the author’s previous works and focuses on searching for novel heat-insulating materials (inspired by bionic structures) produced using 3D additive manufacturing technology. The aim of the present research was to design and make 3D bionic structures based on TBMS structures, and also to test their thermal properties. The research focused on a gyroidal structure, and its ability to reproduce the structure of the inside of a bone to a satisfactory degree. The basic formula describing the gyroid has the form of Equation (1) [7,9,30].

t = sin (x) cos (y) + sin (y) cos (z) + sin (z) cos (x)(1)

When the equation was entered into a parametric modeling program, the standard appearance of a TPMS was obtained. However, gyroidal structures can be infinitely modified by introducing coefficients into the basic equation. In study [30], it was proposed to expand the formula for the gyroid with additional variables

t = [sin (2π·x/a)·cos (2π·y/b)] + [sin (2π·y/b)·cos (2π·z/c)] + [sin (2π·z/c)·cos (2π·x/a)](2)

**Figure 2:**
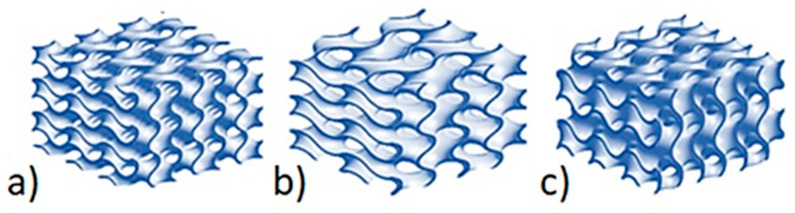
Effect of changes in the value of a, b and c on the shape of the gyroid structure: (**a**) the size of the cell (a = b = c = 2π); (**b**) the size of the cell (a = b = 3π and c = 2π); (**c**) the size of the cell (a = b = 2π and c = 3π).

Variables a, b, and c control the size of the cell in direction x, y, and z, respectively, as shown in Figure 2.

**Figure 3:**
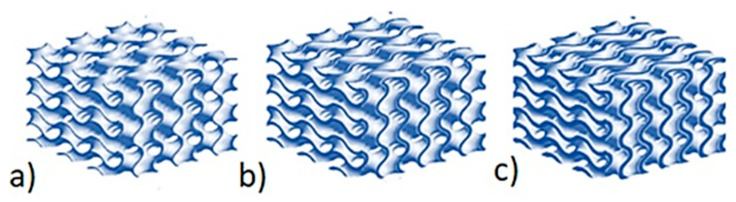
Effect of changes in the value of t on the shape of the gyroid structure: (**a**) wall thickness (t = 0.2), (**b**) wall thickness (t = 0.6), (**c**) wall thickness (t = 1.0).

In Equation (2), parameter t, which is responsible for the ratio of the volumes sepa-rated by the TPMS structure’s surface, can be found. The ratio controls the gyroid’s wall thickness. Wall thickness can also be seen to be one of the parameters that have a significant effect on the heat-insulating properties of an object. A wall that is too thick can increase the share of the insulation’s constant component in the heat transfer, which is a disadvantageous outcome. The effect of a change in the value of parameter t on wall thickness is shown in Figure 3.

**Figure 4:**
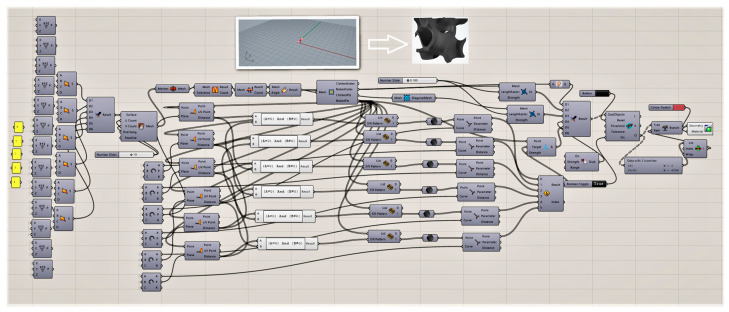
Grasshopper algorithm for creating gyroidal structures.

Rhino 7 with the Grasshopper plugin was used as the parametric design support software (Figure 4). The Grasshopper environment is characterized by the use of a visual programming language, which consists of a graphical and symbolic representation in the form of components/blocks of geometric data, and also operations which can be performed on the data. By putting together appropriate components/blocks, a script reflecting the inter-parameter relations that are described by means of parameters can be built. When designing the internal structure of insulation, this system can be used to look for new forms (form-finding) and attractive designs, or even as a record of an individualized complex geometry [31].

### 2.1. Design and 3D Printing of Multilayer Insulation

The aim of this research was to investigate a bionic bone structure with regards to its thermal conduction. Prototype insulation partitions with a gyroidal structure, which differed in terms of wall thickness t (t = 0.2; 0.6; 1.0 mm) and cell size a, b, and c (a, b, c = 2π or a, b = 3π c = 2π or c = 3π a, b = 2π), were generated. The prototype plates had dimensions of 50 × 50 × 20 mm. Each of the variants was generated as a three-variant set of the number of construction layers: n = 1, 2, 3. Ultimately, 27 prototype plate variants were generated. An SLS 3D printer was used to print the designed models of the prototype insulating material. PA-12 powder (polyamide) was used as the printing material.

The PA-12 powder that was used for the production of the composites is widely applied in many industries. It is a polyamide powder that allows for the printing of prototypes or end parts with high mechanical, chemical, and thermal resistance. Moreover, it can be easily processed and glued, and is characterized by the perfect bonding of layers and a processing shrinkage of below 1.5%. It is an ideal material for producing industrial details such as: sleeves, bearings, screws, or gears, which is due to its high abrasion resistance and high impact strength [32].

It has insulating properties and chemical resistance to greases and oils. Moreover, it has the lowest hygroscopicity out of all the polyamides that are used for 3D printing. The working temperature of ready-made elements may range from 130–150 °C. The printed thin-layered elements have elastic properties. The recommended 3D printing temperature is between 230 and 280 °C. The material is biocompatible and can come into contact with food [33]. The PA 12 material can be used in the following: the construction industry, the transport industry, the food industry, the automotive industry, electrical engineering, and machine elements (bearing sleeves, bushings, gaskets, guide rails).

**Figure 5:**
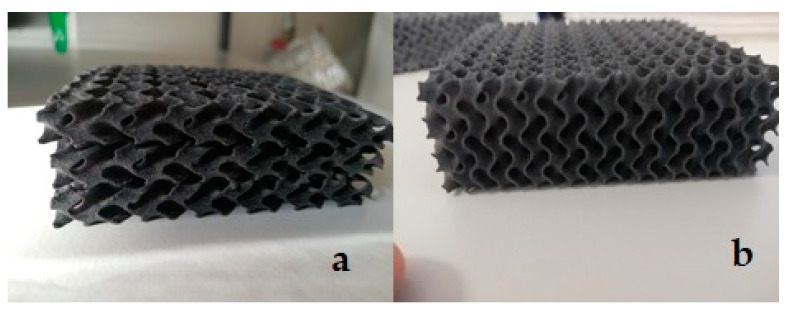
Printing result in the form of ready to use prototype plates: (**a**) three-layer sample (n = 3); (**b**) single-layer sample (n = 1).

The 3D printed heat insulating material is shown in Figure 5.

### 2.2. Experiments

**Figure 6:**
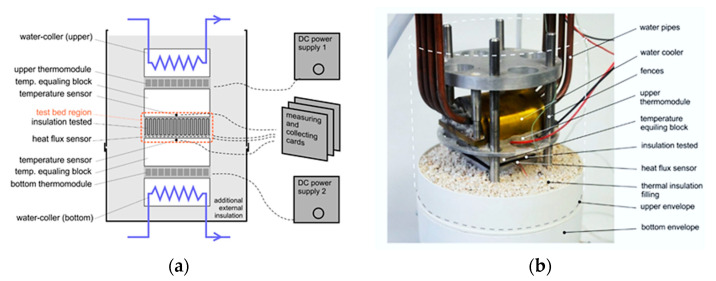
The Poensgen apparatus used by the author: (**a**) schematic diagram, (**b**) photo of the Poensgen apparatus [1,26,27,28].

For each of the described and printed prototype materials that have a gyroid structure, the values of the thermal conductivity coefficient and thermal resistance were determined experimentally. Measurements were taken on a dedicated stand at the Laboratory of Thermal Materials and Devices at the Faculty of Mechanical and Power Engineering at Wroclaw University of Science and Technology. This stand included a thermoelectric version of the Poensgen apparatus, the schematic diagram of which is described below and presented in Figure 6a,b [1,26,27,28].

The Poenseng apparatus measures the heat flux density by creating a temperature difference between the two surfaces of the tested material. In this way, the heat flow is forced, and then detected by the heat flux converter, with the data being recorded on the recording device. The apparatus consists of two parts: one stationary and one pressing element. Both of these elements contain a Peltier module, which enables the temperature of their surfaces to be controlled. Additionally, the stationary element has a heat flux density converter. Controlling the operation of thermocouples involves connecting them to an external DC voltage source. The Peltier module, under the influence of electric current, is able to create a temperature gradient between its surfaces.

Which surface is cooled and which is heated depends on the polarity of the power supply. This means that with the Poenseng apparatus in question, it is possible to choose whether the cooling effect will be exerted on the sample from above or below. Information about the temperatures of the lower and upper walls of the tested sample is provided by two thermocouples that are connected to the recorder. Heat exchange with the environment is minimized by putting a metal sleeve on the apparatus and filling the space around the sample with perlite a granulate of volcanic origin that has a low bulk density and which is characterized by a heat conduction coefficient within the range of 0.045–0.065 W/(m × K).

Experimental measurements were made for two directions of heat flow. In the con-ducted experiment, two methods of heat conduction through the prototype insulation material were taken into account. The first was cooling in the top mode, and for this variant the apparatus was switched to the bottom heating mode with cooling using the top thermomodule. The second type was cooling in the bottom mode, and for this variant the apparatus was switched to the top heating mode with cooling using the bottom thermomodule. The measurements required the establishing of a thermal equilibrium, with the temperature of the heated and cooled side of the device being kept at +20 °C for the hot side and −20 °C for the cold side (fluctuations < 0.1 °C). Based on the measured values, the values of the thermal conductivity and thermal resistance coefficients were calculated for the samples [1,26,27,28,29]. Both values were assumed because of typical working condition of thermal insulation of buildings, food industry, and frozen food transport.

## 3. Results

The experiment was performed according to the full factorial plan. This enabled the main effects and all the possible interactions (including those of the highest order) to be determined.

The experiment was carried out using a complete design for four input factors of 54 sets of five replicates. Statistical analyses were carried out using the tools available in the STATISTICA 13 software (TIBCO Statistica, Palo Alto, USA). A significance level of *p* ≤ 0.05 (this value is usually used in thermal insulation investigations) was assumed in all the analyses.

For the obtained values of the experimental data, the position and dispersion measures were first determined, and their collective results are presented in Table 1.

The values of thermal conductivity coefficient λ ranged from 0.033 to 0.090 W/(m × K) at the mean of 0.064 W/(m × K) and the standard deviation of 0.014 W/(m × K). Half of the tested samples had a result of 0.065 W/(m × K) or less. On the thermal resistance scale, the results were found to be within the interval of 0.221–0.606 W/m^2^K, and the mean amounted to 0.324 W/m^2^K at the deviation of 0.077 W/m^2^K. Half of the samples had a result of 0.310 W/m^2^K or less. The high skewness and kurtosis indicated that the results for most of the samples were low and concentrated around the mean.

**Figure 7:**
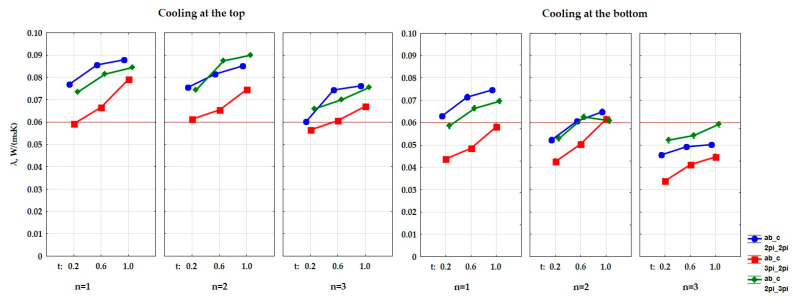
Graphical interpretation of the experimental data that determine the influence of the input factors (independent variables) and their mutual interactions on the value of the thermal conductivity coefficient of a composite with a gyroid structure.

**Figure 8:**
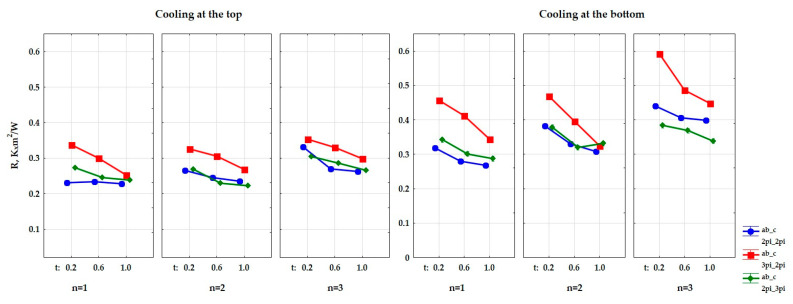
Graphical interpretation of the experimental data that determine the influence of the input factors (independent variables) and their mutual interactions on the value of the thermal resistance coefficient of a composite with a gyroid structure.

Afterwards, it was determined whether the influence of the input quantities on the output quantity is significant. Four factor ANOVA analysis of variance (which includes interactions of up to the fourth-order) was used to determine this impact. The obtained results are presented in Table 2 and Table 3 and in Figure 7 and Figure 8. The presented values of the significance level p, which are lower than 0.05 (the last column of the table), show a significant influence of the type of used cooling, the size of air cells in the composite structure (a, b, c), the thickness of the matrix in the composite (t), and the number of layers in the composite (n) on the value of the thermal conductivity coefficient and thermal resistance of the tested composites. The dependence between the thermal conductivity coefficient (λ) and the kind of cooling, the size of air cells in the composite’s structure (a, b, c), the thickness of the matrix in the composite, and the number of construction layers was determined. It was also determined whether this dependence was interactive. The same was done for thermal resistance (R).

The results of the analysis of variance (Table 2 and Table 3) showed that there was an influence of the type of cooling, the size of the cells (a, b, c), the thickness of the composite matrix (t), and the composite layering (n) on the obtained value of the thermal conductivity coefficient (λ) and the value of the coefficient of thermal resistance (R).

The interdependence of the input factors on the obtained values of thermal properties was analyzed by specifying the multiple regression equation. For this purpose, the method of progressive stepwise regression was used. It examines the significance of the next introduced variable, and also tests a different ordering of the variables at each step. The final model presents the optimal subset of the explanatory variables. The obtained dependencies have the form indicated below. In the case of the thermal conductivity coefficient:

- for the thermal conductivity coefficient:
  λ = −1.829 + 0.019cooling + 0.015t − 0.006n(3)

The obtained model explains about 75% of the variance of the independent variable, with the standard error of the estimation being equal to 0.007.

In the case of the coefficient of thermal resistance:

- for the coefficient of thermal resistance:
  R = 10.505 − 0.101cooling + 0.034n − 0.079t(4)

The obtained model explains about 67% of the variance of the independent variable, with the standard error of the estimation being equal to 0.045.

**Figure 9:**
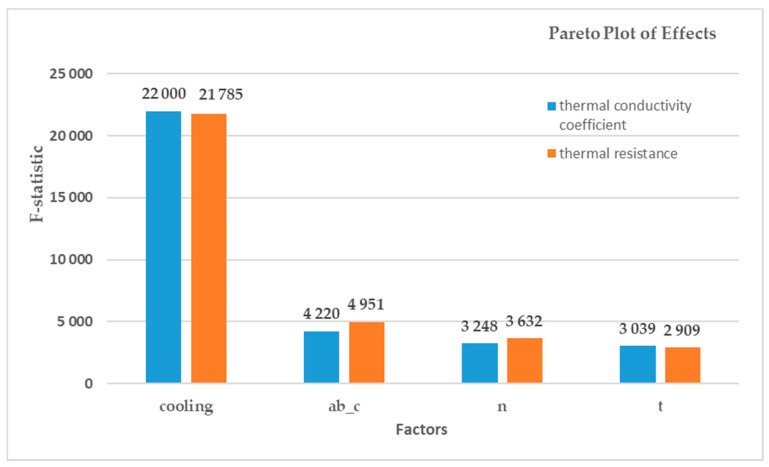
Impact of particular factors on the thermal conductivity (λ) and the thermal resistance coefficient (R).

The analysis also showed a statistical significance of the interaction of linear factors. In order to determine the main dominants, ranking was performed according to the strength of the influence (F) of individual factors and their interaction on the model. The results are presented in Table 4 and Table 5 and in the Pareto chart in Figure 9.

It was shown that the type of cooling applied during the experiment (with a different location in relation to the sample) is a highly dominant factor (BotCool—the bottom-cooled or Top-Cool—the top-cooled) in relation to the other input factors. As shown above, the influence of the best interaction of the input factors (ab_c*n) on the thermal conductivity coefficient is 20 times weaker than that of the worst single input factor (t). Input factor interactions are of little importance with respect to linear factors—i.e., there is the best type of composite structure for each input factor that is not determined by other factors. Therefore, in order to determine the optimization of the composite’s composition, the first four dominant factors were taken into account. To sum up, for the best insulating properties of the prepared composites, there is: the best arrangement of the composite in relation to the direction of the heat flow (cooling of a sample – from the top or bottom), the best values in the case of the size of air cells (a, b, c) and wall thickness (t) in the gyroid structure, and the best value for the number of layers of gyroid structures in the composite. Each input factor is optimized independently of the others.

**Figure 10:**
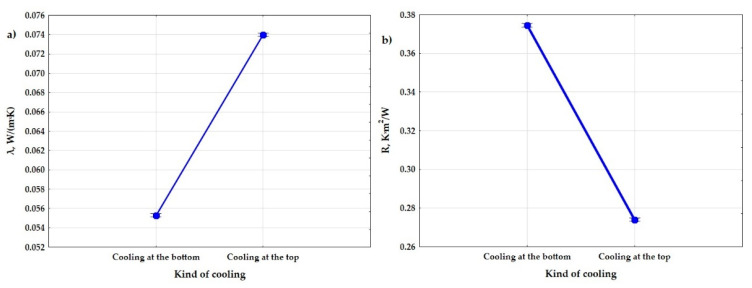
Graphs of the dependence of the thermal conductivity coefficient (**a**) and the thermal resistance coefficient (**b**) from input factor: type of cooling.

The results of the variance analysis, which are presented in Table 4 and Table 5, showed that there is an influence of the type of cooling on the obtained values of the thermal conductivity coefficient and the thermal resistance coefficient. Figure 10 show a graphical comparison of the obtained results for the types of cooling that were used in the experiment. The bottom-cooled samples had a lower thermal conductivity, and thus a higher thermal resistance than the top-cooled samples, and the difference in the obtained results was large. This proves that the composite that was arranged in the direction of heat flow from the top to the bottom had better insulating properties.

**Figure 11:**
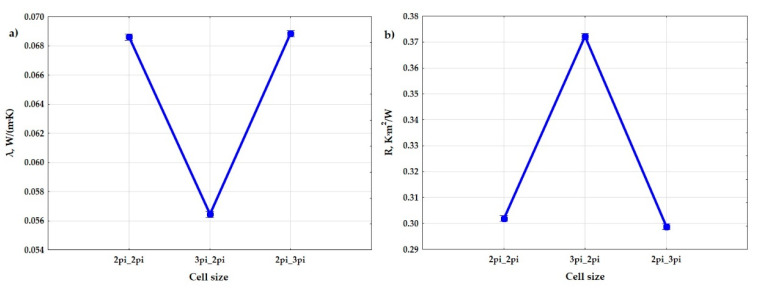
Graphs of the dependence of the thermal conductivity coefficient (**a**) and the thermal resistance coefficient (**b**) from input factor: size of air cells (a,b_c).

It was shown that shape has an influence on the heat conduction coefficient and the heat resistance. Figure 11 show the obtained difference between the parameters of the size of air cells in the gyroid structure. The samples with shape ab_c = 3pi_2pi had a lower coefficient of thermal conductivity (λ), and thus a higher coefficient of thermal resistance, than the samples with shape ab_c = 2pi_2pi and ab_c = 2pi_3pi. The difference in the obtained values was in this case large.

**Figure 12:**
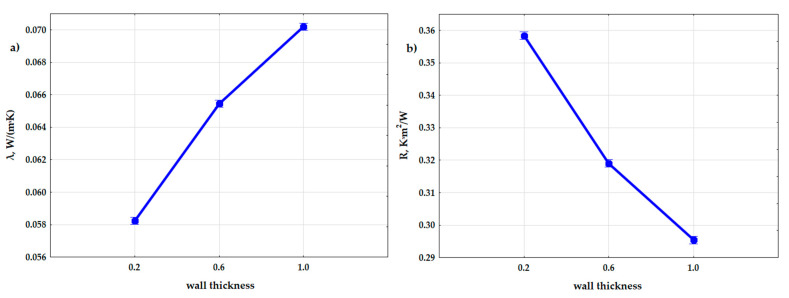
Graphs of the dependence of the thermal conductivity coefficient (**a**) and the thermal resistance coefficient (**b**) from input factor: thickness of the wall in the composite (t).

The results of the variance analysis also proved that shape (in relation to the t di-mension)—i.e., the wall thickness of the gyroid structure, has an influence on the obtained values of the thermal conductivity coefficient (λ) and thermal resistance. Figure 12 shows the relationship between the wall thicknesses in the gyroid structure, which were assumed in the experiment, and the obtained data. The samples with the wall thickness of t = 0.2 mm had a lower thermal conductivity coefficient (λ), and thus a higher thermal resistance, than the samples with t = 0.6 and the samples with t = 1.0, and the difference in the obtained results was moderately large.

**Figure 13:**
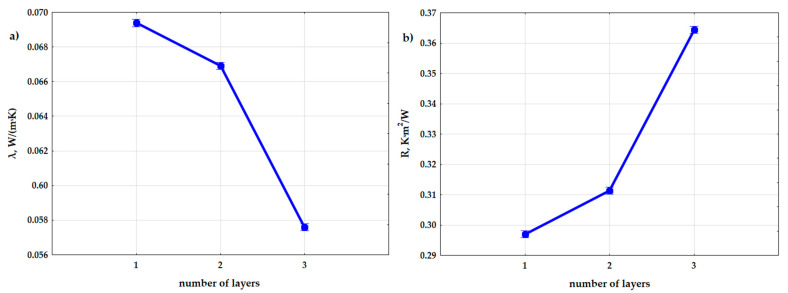
Graphs of the dependence of the thermal conductivity coefficient (**a**) and the thermal resistance coefficient (**b**) from input factor: number of layers (n).

Moreover, the effect of the number of layers (n) in the composite on the obtained values of the thermal conductivity coefficient and the thermal resistance coefficient was also demonstrated during the analysis. Figure 13 show a graphical comparison of the obtained results for the different number of layers that were used in the experiment. The samples with the number of layers n = 3 had a lower thermal conductivity coefficient, and thus a higher thermal resistance, than the samples with n = 2 and the samples with n = 1. The difference in the obtained results was moderately large.

**Figure 14:**
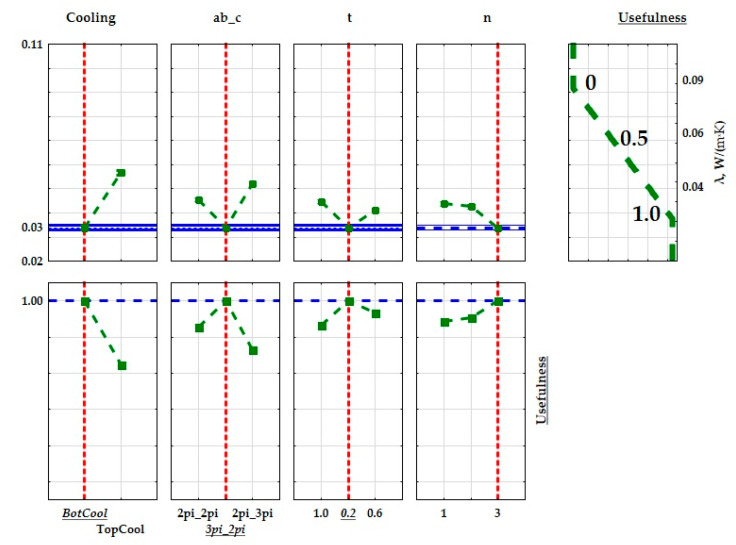
Graphical interpretation of the optimization of the composite structures with regards to the determined thermal conductivity coefficient (λ).

**Figure 15:**
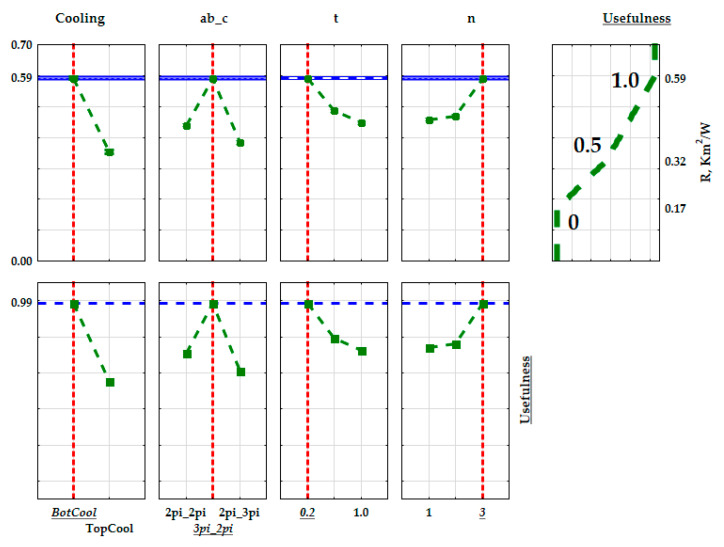
Graphical interpretation of the optimization of the composite structures with regards to the thermal resistance coefficient (R).

In order to optimize the structure of the created composites so as to obtain the best thermal properties of the insulating materials to be used in construction and industry, the lower, intermediate, and upper values were calculated for the determined thermal conductivity and thermal resistance coefficients. Usefulness values were assigned to the lower and upper values. When maximizing the value of a given parameter (the max criterion), the upper value was assigned the usefulness value of 1.0, while the lower value was assigned the usefulness value of 0. As usefulness values change linearly between these bounds, the intermediate value was assigned the usefulness value of 0.5. The extreme mean values of the thermal conductivity coefficient and the thermal resistance coefficient are presented on in Figure 14 and Figure 15. Due to the fact that the values of the thermal conductivity coefficient are relatively uniform, the lowest value of the thermal conductivity coefficient and the highest value of thermal resistance were specified as the criteria for determining the best insulating properties of the produced composite. The most useful material had a structure with the best insulating thermal properties.

## 4. Conclusions

On the basis of the multicriteria analysis, the composite’s optimal composition according to the adopted optimization criteria was determined. The lowest thermal conductivities were obtained for the prototype insulation partitions with a gyroidal structure, which had: the wall thickness t = 0.2 mm, cell size a, b and c (at a,b = 3π and c = 2π), the number of construction layers n = 3, and cooling from the bottom. The lowest possible thermal conductivity of the insulation was equal to 0.033 W/(m·K), and the highest possible thermal resistance was equal to 0.606 m^2^·K/W. The highest thermal conductivity coefficient, and thus the lowest thermal resistance were obtained for a composite that had: the wall thickness t = 1 mm, cell size a, b, and c (at a,b = 2π and c = 3π), the number of construction layers n = 1 and cooling from the top. According to PN-EN ISO 9229: 2020-12, thermal insulating materials achieve a thermal conductivity coefficient of no higher than 0.065 W/(m × K). By analyzing the results, it was possible to obtain the desired value of the thermal conductivity coefficient for most of the samples. The prototype insulation partition with a gyrodal structure is characterized by good insulation parameters. Thanks to the statistical analysis of the experimental results, the composition of the composite could be optimized in accordance with the adopted criteria.

The performed tests, which determined the thermal properties of the obtained prototype structures, prompted the author to carry out the next stage of research, the purpose of which is to determine the mechanical and strength properties of the obtained structures. A very interesting issue seems to be the use of prototypical gyroid structures (filled with air) for plastic window frames [1] to be used in the construction industry, as well as the use of these structures as a material for collective packaging for frozen food products, which is the author’s interest and a continuation of the already started research.

## Figures and Tables

**Table 1:** Descriptive statistics for the thermal conductivity coefficient (λ) and thermal resistance (R) (Min—minimum; Max—maximum; M—mean; SD—standard deviation; Me—median; Sk—skewness; K—kurtosis).

	Min	Max	M	SD	Me	Sk	K
λ, W/(m × K)	0.033	0.090	0.064	0.014	0.065	−0.071	−0.726
R, W/m^2^K	0.221	0.606	0.324	0.077	0.310	1.080	1.303

**Table 2:** Quantitative assessment of the main effects and the effects of interactions—identification of the impact of dominant and statistically significant input factors on the dependent variable λ.

Symbol That Identifies the Input Factors and Their Interactions	SS	df	MS	F	*p*
absolute term	1.127792	1	1.127792	1,052,144	0.000
cooling	0.023574	1	0.023574	21,993	0.000
abc	0.009047	2	0.004523	4220	0.000
t	0.006516	2	0.003258	3039	0.000
n	0.006962	2	0.003481	3248	0.000
cooling*ab_c	0.000006	2	0.000003	3	0.052
cooling*t	0.000040	2	0.000020	18	0.000
ab_c*t	0.000383	4	0.000096	89	0.000
cooling*n	0.000318	2	0.000159	148	0.000
ab_c*n	0.000749	4	0.000187	175	0.000
t*n	0.000113	4	0.000028	26	0.000
cooling*ab_c*t	0.000046	4	0.000012	11	0.000
cooling*ab_c*n	0.000628	4	0.000157	146	0.000
cooling*t*n	0.000051	4	0.000013	12	0.000
ab_c*t*n	0.000228	8	0.000029	27	0.000
cooling*ab_c*t*n	0.000249	8	0.000031	29	0.000
error	0.000232	216	0.000001		

**Table 3:** Quantitative assessment of the main effects and the effects of interactions—identification of the impact of dominant and statistically significant input factors on the dependent variable R.

Symbol That Identifies the Input Factors and Their Interactions	SS	df	MS	F	*p*
absolute term	28.38911	1	28.38911	907,188.5	0.000
cooling	0.68172	1	0.68172	21,784.7	0.000
abc	0.30987	2	0.15494	4951.1	0.000
t	0.18208	2	0.09104	2909.2	0.000
n	0.22732	2	0.11366	3632.1	0.000
cooling*ab_c	0.02900	2	0.01450	463.3	0.000
cooling*t	0.01285	2	0.00642	205.3	0.000
ab_c*t	0.03254	4	0.00814	260.0	0.000
cooling*n	0.03363	2	0.01682	537.4	0.000
ab_c*n	0.02592	4	0.00648	207.1	0.000
t*n	0.00174	4	0.00044	13.9	0.000
cooling*ab_c*t	0.00779	4	0.00195	62.2	0.000
cooling*ab_c*n	0.02338	4	0.00584	186.7	0.000
cooling*t*n	0.00106	4	0.00026	8.5	0.000
ab_c*t*n	0.00676	8	0.00084	27.0	0.000
cooling*ab_c*t*n	0.00987	8	0.00123	39.4	0.000
error	0.00676	216	0.00003		

**Table 4:** Result of the conducted ranking, which took into account the effect of the value that determines the strength of the influence (F) of the input factors and their interactions on the determined values of the thermal conductivity coefficient.

Rank	Symbol That Identifies the Input Factors and Their Interactions	SS	df	MS	F	*p*
1	cooling	0.024	1	0.024	22,000	0.000
2	ab_c	0.009	2	0.005	4220	0.000
3	n	0.007	2	0.003	3248	0.000
4	t	0.007	2	0.003	3039	0.000
5	ab_c*n	0.001	4	0	175	0.000
6	cooling*n	0	2	0	148	0.000
7	cooling*ab_c*n	0.001	4	0	146	0.000
8	ab_c*t	0	4	0	89	0.000
9	cooling*ab_c*t*n	0	8	0	29	0.000
10	ab_c*t*n	0	8	0	27	0.000
11	t*n	0	4	0	26	0.000
12	cooling*t	0	2	0	18	0.000
13	cooling*t*n	0	4	0	12	0.000
14	cooling*ab_c*t	0	4	0	11	0.000
15	cooling*ab_c	0	2	0	3	0.052

**Table 5:** Result of the conducted ranking, which took into account the effect of the value that determines the strength of the influence (F) of the input factors and their interactions on the determined values of the thermal resistance coefficient.

Rank	Symbol That Identifies the Input Factors and Their Interactions	SS	df	MS	F	*p*
1	cooling	0.68172	1	0.68172	21,784.7	0.000
2	ab_c	0.30987	2	0.15494	4951.1	0.000
3	n	0.22732	2	0.11366	3632.1	0.000
4	t	0.18208	2	0.09104	2909.2	0.000
5	cooling*n	0.03363	2	0.01682	537.4	0.000
6	cooling*ab_c	0.029	2	0.0145	463.3	0.000
7	ab_c*t	0.03254	4	0.00814	260	0.000
8	ab_c*n	0.02592	4	0.00648	207.1	0.000
9	cooling*t	0.01285	2	0.00642	205.3	0.000
10	cooling*ab_c*n	0.02338	4	0.00584	186.7	0.000
11	cooling*ab_c*t	0.00779	4	0.00195	62.2	0.000
12	cooling*ab_c*t*n	0.00987	8	0.00123	39.4	0.000
13	ab_c*t*n	0.00676	8	0.00084	27	0.000
14	t*n	0.00174	4	0.00044	13.9	0.000
15	cooling*t*n	0.00106	4	0.00026	8.5	0.000

## Data Availability

Data sharing not applicable.

## Nomenclature

R	thermal resistance, m^2^·K/W
Min	minimum
Max	maximum
M	mean
SD	standard deviation
Me	median
Sk	skewness
K	kurtosis
SS	sum-of-squares
MS	mean square
df	degrees of freedom
F	F ratio
*p*	significance level (*p*-values)
a,b,c	the size of the gyroid’s air cells
t	wall thickness of the solid phase of the gyroid structure
n	the number of construction layers in the composite
coolig	the input factor that determines all the types of cooling applied in the experiment
abc	the input factor that defines all the assumed modifications of the size of cells in the structure of the gyroid
cooling*ab_c	the interaction between the applied cooling, the size of air cells in the gyroid structure and the values of the output data obtained in the experiment
cooling*t	the interaction between the applied cooling, the wall thickness of the gyroid structure and the values of the output data obtained in the experiment
ab_c *t	the interaction between the size of cells, the wall thickness of the gyroid structure and the values of the output data obtained in the experiment
cooling *n	the interaction between the applied cooling, the layering of the composite with the gyroid structure and the values of the output data obtained in the experiment
ab_c*n	the interaction between the size of air cells in the gyroid structure, the layering of the composite with the gyroid structure and the values of the output data obtained in the experiment
t*n	the interaction between the wall thickness in the gyroid structure, the layering of the composite with the gyroid structure and the values of the output data obtained in the experiment
cooling*ab_c*t	the interaction between the applied cooling, the size of air cells, the wall thickness in the gyroid structure and the values of the output data obtained in the experiment
cooling*ab_c*n	the interaction between the applied cooling, the size of air cells in the gyroid structure, the layering of the composite and the values of the output data obtained in the experiment
cooling*t*n	the interaction between the applied cooling, the wall thickness of the gyroid structure, the layering of the composite and the values of the output data obtained in the experiment
ab_c*t*n	the interaction between the size of air cells, the wall thickness of the gyroid structure, the layering of the composite and the values of the output data obtained in the experiment
cooling*ab_c*t*n	the interaction between the applied cooling, the size of air cells, the wall thickness of the gyroid structure, the layering of the composite and the values of the output data obtained in the experiment.
Greek symbols	
λ	thermal conductivity coefficient, W/(m·K)
